# The new landscape of *APOE* genotyping in the Alzheimer’s Clinic: Harnessing genetic counseling experience to create digital tools to support patients considering anti‐amyloid therapies

**DOI:** 10.1002/alz.089364

**Published:** 2025-01-09

**Authors:** Elisabeth Wood, Cara Cacioppo, Michelle Weinberg, Laynie Dratch, Meron Azage, Rajia Mim, Demetrios Ofidis, Sarah Milinski, Carolyn Langlois, Kristin Harkins, Claire M Erickson, Brian L Egleston, Jason Karlawish, Jessica B. Langbaum, Angela R. Bradbury

**Affiliations:** ^1^ University of Pennsylvania, Philadelphia, PA USA; ^2^ Banner Alzheimer’s Institute, Phoenix, AZ USA; ^3^ Fox Chase Cancer Center, Philadelphia, PA USA

## Abstract

**Background:**

With the advent of FDA approved anti‐amyloid therapy and recognition of increased side effects in *APOE* e4 carriers, *APOE* testing is now recommended for patients considering anti‐amyloid therapies such as lecanemab. Given the therapeutic implications and anticipated volume of eligible patients, the traditional model of in‐person, pre‐ and post‐test genetic counseling is not feasible to incorporate in clinical pathways. Alternative delivery models, including digital tools and telehealth, will be key in providing *APOE* genetic counseling support.

**Methods:**

The Penn Telegenetics Program provided *APOE* genotype disclosure to cognitively intact participants across the United States in two Alzheimer’s prevention studies (API Generation Studies 1 and 2). An ancillary, multi‐site randomized study (CONNECT 4 APOE) evaluated the relative advantages of real time videoconference over telephone for disclosure of *APOE* genotype results within the API Generation Program. Outcomes of these studies informed the development of digital tools to support genetic counseling and educational needs specific to this patient population. Standard user and usability testing as well as rapid cycle testing were used in the development of digital tools.

**Results:**

Over 2600 API Generation Program participants received *APOE* genotype disclosure via the Penn Telegenetics Program, and over 600 participants enrolled in the ancillary CONNECT 4 APOE protocol. This work informed the development of clinical chatbots for patients undergoing *APOE* testing for anti‐amyloid therapy. These chats include an education chat for patients prior to receiving *APOE* results and an *APOE* result specific chat to provide support to patients after receipt of results. Chatbot development and results of rapid cycle testing with patients will be described. Additionally, experiences with initial implementation of these digital tools and requests for genetic counseling among patients and their relatives will be reported.

**Conclusions:**

Harnessing the experiences and outcomes from the described studies provides a unique opportunity to better understand use of telehealth modalities for *APOE* genotype disclosure and launch the development of digital tools that can further support the genetic counseling needs of this patient population. As indications for APOE testing rapidly increase, alternative delivery models will be critical to maximizing benefits and limiting harms.

